# Cardiovascular risk in US adults with nonalcoholic steatohepatitis (NASH) vs. matched non-NASH controls, National Health and Nutrition Examination Survey, 2017–2020

**DOI:** 10.1371/journal.pone.0309617

**Published:** 2024-08-27

**Authors:** Jonathan J. Woolley, Jesse Fishman, Christina M. Parrinello, Tom O’Connell

**Affiliations:** 1 Medicus Economics, Boston, Massachusetts, United States of America; 2 Formerly of Madrigal Pharmaceuticals, Conshohocken, Pennsylvania, United States of America; 3 Pine Mountain Consulting, Redding, Connecticut, United States of America; Universitatsklinikum Leipzig, GERMANY

## Abstract

**Background:**

NASH is considered a contributor to atherosclerotic cardiovascular disease (ASCVD) risk; however, its contribution beyond traditional risk factors for CVD, particularly diabetes, is less clearly understood. This study aimed to quantify the cardiovascular-event risk associated with NASH, independent of diabetes status.

**Methods:**

A cross-sectional analysis was conducted using the 2017–2020 NHANES pre-pandemic cycle. NASH was defined based on presence of steatosis without other causes of liver disease, and FibroScan+AST score from vibration-controlled transient elastography (VCTE). Significant fibrosis (stages F2-F4) was identified by liver stiffness measurement from VCTE. Predicted primary CV-event risk was estimated using both the Pooled Cohort Equations (PCE) and the Framingham Risk Score (FRS). NASH patients were matched with non-NASH controls on age, sex, race/ethnicity, and diabetes status. Weighted logistic regression was conducted, modeling elevated predicted CV risk (binary) as the dependent variable and indicators for NASH / fibrosis stages as independent variables.

**Results:**

A sample of 125 NASH patients was matched with 2585 controls. NASH with significant fibrosis was associated with elevated predicted 10-year CV risk, although this association was only statistically significant in PCE analyses (odds ratio and 95% CI 2.34 [1.25, 4.36]). Analyses restricting to ages <65 years showed similar results, with associations of greater magnitude.

**Conclusion:**

Independent of diabetes, a significant association was observed between NASH with significant liver fibrosis and predicted primary CV-event risk in US adults, particularly for those <65. These findings suggest the importance of accounting for NASH and liver-fibrosis stage in predicting CV-event risk.

## Introduction

Non-alcoholic fatty liver disease (NAFLD) is a condition believed to affect more than 25% of adults worldwide, [1] in which excess fat is stored in the liver (hepatic steatosis), exceeding 5%-10% of the liver’s weight. NAFLD is not attributable to other causes of chronic liver disease [2, 3] further classified into non-alcoholic fatty liver (NAFL) and non-alcoholic steatohepatitis (NASH). NASH is distinguished from NAFL by presence of inflammation and hepatic injury [2, 3]. It has been estimated that approximately 20%-30% of people with NAFLD have NASH [4].

NASH is an increasingly significant public health concern in the US [5]. Current estimates of NASH in US adults suggest a prevalence of 5% to 6%, [6–8] but it has been observed to range from approximately 1% [9] to 12% [10]. The prevalence has risen over the past decade, [9] and is expected to continue to increase substantially in future [11]. Progression of NASH is recognized to lead to cirrhosis, followed by potential decompensation (i.e., liver failure) and hepatocellular carcinoma (HCC) [12].

Beyond the liver-related complications of NAFLD/NASH, increasing evidence has emerged in recent years of the association with multisystem disease affecting a variety of extra-hepatic organ systems, including cardiovascular [13, 14]. NAFLD is recognized to be a risk factor for atherosclerotic cardiovascular disease (CVD), which is a principal cause of death in NAFLD patients [13]. Although the mechanism for the relationship between CVD and NAFLD remains under investigation, the pathophysiology associated with inflammation in fibrotic NASH may exacerbate insulin resistance and dyslipidemia in tandem with a release of proinflammatory, vasoactive, and thrombogenic factors that may promote the development of CVD [6].

The association of NAFLD/NASH and CVD is recognized in a recent scientific statement by the American Heart Association (AHA), [1] emphasizing that NASH enhances cardiovascular risk. The AHA notes that research has demonstrated that NASH has an incremental adverse impact on atherosclerotic cardiovascular disease (ASCVD) risk, beyond traditional risk factors. However, NASH is currently categorized as a risk enhancer, as the AHA statement notes that the risk-modulating effect has not been quantified numerically.

Growing evidence suggests that NAFLD may both increase the risk of incident T2D by exacerbating hepatic insulin resistance, and worsen glycemic control in patients with T2D, contributing to risk of progression of T2D to important chronic complications such as CVD and chronic kidney disease [15]. T2D is considered a major risk factor for CVD, [16, 17] and to be bi-directionally associated with NAFLD, [15] potentially confounding the estimation of CVD risk associated with NASH.

A related, additional challenge in numerical quantification of excess CV-event risk associated with NASH is that current CV-risk prediction equations do not account for possible risk factors involved in the relationship between NASH and CVD. As noted by the AHA, while risk equations such as the Pooled Cohort Equations and Framingham Risk Score are commonly used in the general population, their utility in persons with NASH is not well-established [1]. The association of NAFLD/NASH with CVD has been observed independently of traditional risk factors, such as those included in these risk equations (i.e., blood pressure, diabetes, and dyslipidemia) [18–21]. For example, body mass index/obesity is not included in current CV-risk prediction equations; however, it has been hypothesized that ectopic fat deposition in the liver (i.e., NAFLD) and other tissues may be associated with increased CVD risk beyond that attributable to traditional risk factors [1]. Additionally, the risk of CVD in NAFLD/NASH has also been shown to be particularly elevated in those with advanced or significant fibrosis, [22–24] although fibrosis is not included in current risk equations.

Estimation of excess CV-event risk associated with NASH, as well as addressing potential confounding of T2D, may help inform clinical understanding of the relationship between NASH and CVD. Accordingly, this analysis sought to estimate the association of NASH with risk of a primary CV event, independent of diabetes status. This analysis was undertaken by estimating the predicted 10-year probability of a first cardiovascular event using published risk equations in persons predicted to have NASH, comparing to that in non-NASH controls, matching on diabetes status and other demographic factors. To explore potential drivers of the association of NASH with CV risk, the incremental CV risk was evaluated for NASH overall as well as by estimated fibrosis stages (no fibrosis or minimal fibrosis vs significant fibrosis).

## Methods

### Data source and study design

This was a cross-sectional study using data from the 2017 to March 2020 pre-pandemic continuous National Health and Nutrition Examination Survey (NHANES) cycle. NHANES data are made publicly available by the National Center for Health Statistics (NCHS) and include participants from the non-institutionalized civilian US population. Sample weights are needed to account for the complex survey design/oversampling, survey non-response, and post-stratification adjustment to align with Census counts. Weighting of the NHANES data is expected to produce estimates representative of the civilian resident non-institutionalized US population.

Due to the COVID-19 pandemic, the 2019–2020 survey cycle was not completed. However, NCHS combined the 2019–2020 partial data with the previous 2017–2018 cycle data to create the 2017 to March 2020 pre-pandemic cycle. This survey cycle covers a 3.2-year period (compared with the previous 2-year survey cycles) and can be weighted to provide nationally representative estimates as in previous survey cycles [25]. In the 2017–2018 cycle, NHANES included for the first time measures of liver stiffness and steatosis from vibration controlled transient elastography (VCTE), which were then supplemented with measures from 2019–2020 in the 2017–2020 cycle. This study therefore used the 2017–2020 NHANES cycle, to leverage additional sample and to improve data recency vs. the 2017–2018 cycle alone. The 2017–2020 NHANES cycle includes survey interview responses for 15,560 participants, of whom 14,300 completed the medical examination.

The NHANES 2017–2018 and 2019–2020 surveys received ethics approval under NCHS Research Ethics Review Board Protocols #2011–17 and #2018–01 [26].

### Study population

**Fig 1:**
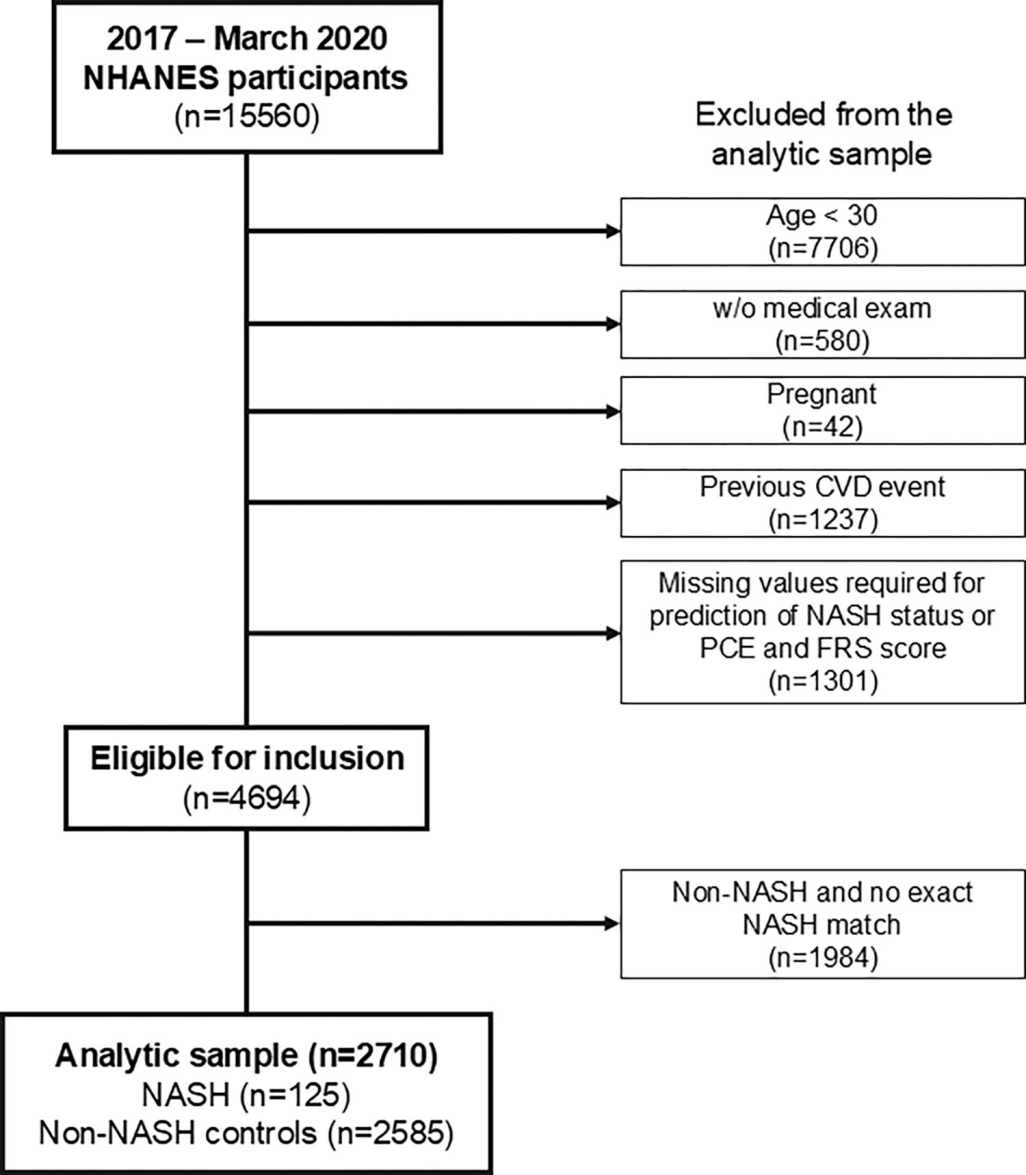
Study population flow diagram. Participants were classified as NASH based on presence of steatosis (liver fat content ≥5% based on CAP ≥302 dB/m), lack of other liver disease (excessive alcohol consumption, hepatitis B, or hepatitis C), and FAST score score ≥0.48. Abbreviations: CAP, controlled attenuated parameter; FAST, FibroScan-aspartate aminotransferase; NASH, nonalcoholic steatohepatitis.

The analytic sample was determined through a combination of complete case variables availability, and either identified as NASH or exact matched as a non-NASH control (Fig 1). Eligibility was restricted to participants who completed the NHANES medical examination, were not pregnant, were ≥30 years of age, had complete data for variables required for prediction of NASH status, had no history of atherosclerotic CVD (ASCVD–defined as CHD, MI, stroke or angina) or heart failure (HF), and had complete data for inputs of the risk equations for a primary CV event. Eligible participants were then restricted to only those identified as NASH and non-NASH exact matched controls for the analytic sample.

### Prediction of NASH

Prediction of NASH was conducted using a multistep approach, similar to other NHANES studies, [27, 28] based on presence of presumed NAFLD, indicated by steatosis (CAP ≥ 302 dB/m) and no alternative common cause of liver disease (i.e., excessive alcohol consumption, hepatitis B or C); [29] and FibroScan-aspartate aminotransferase (FAST) score ≥0.48, to predict NASH among those with presumed NAFLD [30]. In a population with biopsy-proven NAFLD, FAST scores ≥0.48 have been reported to predict definite NASH (NAFLD Activity Score [NAS] ≥5) with area under the receiver operating characteristic curve (AUC) of 0.752, sensitivity of 80.3%, specificity of 58.2%, positive predictive value (PPV) of 62.7%, and negative predictive value (NPV) of 77.2% [30]. This threshold also reflects the approximate midpoint of the rule-out (≤0.35) and rule-in (≥0.67) cutoffs reported for the FAST score for predicting at-risk NASH (NAS ≥4 and fibrosis stage ≥2) in patients with suspected NAFLD, [31] suggesting that the sample predicted to have NASH in this analysis may lean towards higher fibrosis stages.

Participants predicted to have NASH were further categorized by estimated fibrosis stage. The following liver stiffness measurement (LSM) thresholds were applied, as reported in Eddowes et al. (2019): [32] <8.2 kPa for F0-F1, 8.2 to <9.7 for F2, 9.7 to <13.6 for F3, and ≥13.6 for F4. Participants with estimated fibrosis stage of F2 or higher (i.e., LSM ≥8.2) were considered to have significant fibrosis, while estimated stages F0-F1 (i.e., LSM <8.2) were considered no/minimal fibrosis.

A multi-society and multi-stakeholder consensus revision of the NAFLD nomenclature occurred with the results as follows: Steatotic Liver Disease (SLD) with sub-classifications of Metabolic dysfunction Associated Steatotic Liver Disease (MASLD) and Metabolic dysfunction Associated Steatohepatitis (MASH) [29]. While developments in the classification of NAFLD/NASH were considered during the conduct of this analysis, to maintain consistency with the context and motivation established by the aforementioned AHA scientific statement, [1] NASH was kept as the exposure of interest in the analysis.

### Prediction of CV risk

The primary analysis calculated the 10-year probability of a primary CV event for each study participant using the 2013 ACC/AHA Pooled Cohort Equations (PCE) [16]. The PCE predicts risk of a first ASCVD event defined as CHD death, nonfatal MI, and both fatal and nonfatal stroke. The following variables are included in the PCE: age, total cholesterol, high-density lipoprotein cholesterol (HDL-c), systolic blood pressure (SBP), SBP treatment, smoking status, and diabetes status.

Sensitivity analyses used the 2008 Framingham Risk Score (FRS) [17]. Although the same variables are included in both the PCE and FRS, the outcomes that are modeled and the form of the equations differ. The FRS predicts risk of a first CV event defined as CHD: coronary death, myocardial infarction, coronary insufficiency, and angina; cerebrovascular events: including ischemic stroke, hemorrhagic stroke, and transient ischemic attack; peripheral artery disease (intermittent claudication); and HF.

The FRS was validated in participants aged 30–74 years of age with no race/ethnicity restrictions, whereas the PCE was validated in non-Hispanic (NH) Black and White participants aged 40–79 years. However, the existing literature suggests that the PCE has good discrimination in Hispanic and Asian populations (even though calibration is limited for all races/ethnicities) [33, 34]. Accordingly, to increase both sample size and generalizability, analyses using the PCE did not restrict race/ethnicity. Additionally, main analyses included participants aged ≥30 years. However, sensitivity analyses were conducted for analyses of the PCE that restricted to NH Black and White participants aged ≥40 years. Because the performance of CV risk equations has been shown to worsen with increasing age, [35] we conducted additional sensitivity analyses for both risk equations that narrow the age range to those <65 years.

### Covariates

In NHANES, the following variables were self-reported by the participant: age, sex, race/ethnicity, current smoking, diabetes (self-reported physician diagnosis), blood pressure treatment, and history of hepatitis B or C.

Various laboratory measurements were used in this analysis for calculation of predicted CV risk and prediction of NASH status. Total cholesterol, triglycerides, alanine transaminase (ALT), and aspartate transferase (AST) were measured using an enzymatic method, HDL-c was measured using the direct method, and platelets were measured from whole blood. LDL-c was calculated using the Friedewald equation [36]. FIB-4 was calculated as age x AST / (platelets x √(ALT)) [37].

Three consecutive blood pressure measurements were taken 60 seconds apart and were averaged (for diastolic and systolic BP, separately) to obtain the measurement used for analysis. Height, weight, and waist circumference were recorded by trained health technicians. BMI was calculated from measured weight and height as kg/m^2^. Obesity was defined as BMI ≥30 kg/m^2^.

### Statistical analysis

Participants identified as having NASH were exact matched to a group of non-NASH controls, based on age groups (30–34, 35–39, 40–44, 45–49, 50–54, 55–59, 60–64, 65–69, 70–74, 75+ years), sex (male, female), race/ethnicity category (non-Hispanic Black, non-Hispanic White, Hispanic, non-Hispanic Asian, non-Hispanic Other), and diabetes status (yes, no).

Matching performance was evaluated by assessing the standardized mean differences (SMD) of the weighted covariates in NASH versus non-NASH participants before and after matching. After matching, all covariates were within the 0.1 recommended SMD threshold, [38] with all except sex within 0.05.

Demographic and clinical characteristics were summarized and compared in participants with NASH and matched non-NASH participants. Weighted proportions (for categorical variables) and weighted means (for continuous variables) with corresponding standard errors were reported. P-values were calculated using survey-weighted chi-square tests with the Rao-Scott correction for categorical variables and survey-weighted t-tests for continuous variables.

Weighted logistic regression was used to evaluate the association between NASH and 10-year predicted CV risk as a binary variable. Ten-year predicted CV risk was defined as elevated if ≥7.5% in analyses using the PCE, and if ≥20% for analyses using the FRS, based on treatment guidelines [39]. NASH (the independent variable of interest) was modeled in two ways: as a binary variable (NASH vs. no NASH) and as a categorical variable (NASH with significant fibrosis, NASH with no/minimal fibrosis, and no NASH [reference group]). Odds ratios and corresponding 95% CIs were obtained. P-values from Wald tests were also calculated. Analyses were conducted overall and restricted to participants aged <65 years. A secondary analysis used linear regression to estimate the association between NASH and 10-year predicted CV risk as a continuous variable.

For all final models, sampling weights and the Taylor series linearization variance approximation method were used to obtain point estimates and corresponding standard errors.

All statistical analyses were conducted using R (version 4.2.0).

### Compliance with ethics guidelines

The analysis reported is based on public-use data and does not contain any new studies with human participants or animals performed by any of the authors. The NHANES 2017–2018 and 2019–2020 protocols were approved by the NCHS Research Ethics Review Board Protocols #2011–17 and #2018–01 and were accessed between March 21, 2023 and December 18, 2023. At no point did the authors access information that could identify individual participants.

## Results

### Matching

An unweighted sample of 125 participants was predicted to have NASH, then exact matched with 2585 non-NASH participants. In the matched sample, compared with participants without NASH, those with NASH were more likely to be obese (85% vs. 42%), hypertensive (65% vs. 42%), have low HDL-c (57% vs. 31%), and were less likely to be a current smoker (7% vs. 18%) (all p < 0.01) (Table 1). As expected, a significantly higher proportion of participants with NASH had significant fibrosis (75% vs. 10%, p < 0.01).

**Table 1:** Characteristics of matched participants with and without NASH.

	Weighted % (SE) or		P-value
	Weighted mean (SE)		
	NASH	Matched^1^	
	(n = 125)	No NASH	
		(n = 2585)	
***Demographics and clinical characteristics***			
Age (years)	48.8 (1.28)	49.3 (0.49)	0.69
Female	28.5% (5.3%)	32.3% (1.5%)	0.49
Race / ethnicity			
Non-Hispanic White	67.4% (5.1%)	67.1% (2.8%)	0.94
Non-Hispanic Black	3.0% (0.8%)	3.5% (0.5%)	0.56
Non-Hispanic Asian	4.8% (1.6%)	4.4% (0.6%)	0.82
Hispanic	17.7% (3.2%)	19.1% (2.2%)	0.70
Non-Hispanic Other	7.1% (3.6%)	5.9% (1.3%)	0.71
Diabetes (self-reported diagnosis)	24.9% (4.9%)	23.2% (1.8%)	0.77
Obese (BMI ≥30 kg/m^2^)	85.2% (3.2%)	41.9% (2.5%)	<0.01
Significant fibrosis (F2-F4)	75.1% (5.3%)	10.5% (1.4%)	<0.01
***CVD risk factors***			
Current smoker	6.7% (2.3%)	18.0% (1.5%)	<0.01
Systolic blood pressure (SBP), mmHg	125.7 (1.22)	122.2 (0.63)	0.01
High SBP (≥ 130 mmHg)	30.2% (5.7%)	24.8% (1.8%)	0.25
On BP medication^2^	33.0% (5.9%)	23.0% (1.7%)	0.09
Hypertension^3^	64.7% (6.8%)	42.0% (2.3%)	<0.01
Total cholesterol, mg/dL	200.0 (4.86)	190.6 (1.66)	0.054
High total cholesterol (≥ 200 mg/dL)	46.6% (6.7%)	39.0% (2.0%)	0.22
HDL-c, mg/dL	41.7 (1.27)	50.7 (0.62)	<0.01
Low HDL-c^4^	56.9% (4.6%)	31.0% (2.2%)	<0.01
LDL-c, mg/dL	113.7 (5.07)	108.0 (2.00)	0.28
High LDL-c (≥ 130 mg/dL)	29.9% (6.1%)	24.3% (1.8%)	0.38
High LDL-c (≥ 100 mg/dL)	62.7% (5.7%)	59.8% (1.9%)	0.59

P-values were calculated using survey-weighted chi-square tests with the Rao-Scott correction for categorical variables and using survey-weighted t-tests for continuous variables

Notes: (1) Participants identified as having NASH were exact matched to a group of non-NASH controls, based on age groups (30–34, 35–39, 40–44, 45–49, 50–54, 55–59, 60–64, 65–69, 70–74, 75+ years), sex (male, female), race/ethnicity category (non-Hispanic Black, non-Hispanic White, Hispanic, non-Hispanic Asian, non-Hispanic Other), and diabetes status (yes, no). (2) Defined as self-reported current use of a prescription for hypertension among participants who reported ever being told by a doctor that they had high blood pressure. (3) Hypertension reflects SBP ≥130, DBP ≥85, or self-report of treatment for high BP. (4) Lower than 50 mg/dL for females and 40 mg/dL for males.

### CV risk

After matching, compared with no NASH, NASH with significant fibrosis was associated with higher odds of having elevated predicted 10-year CV risk, although this association was only statistically significant for the PCE and not the FRS (PCE: OR [95% CI]: 2.34 [1.25, 4.36], and FRS: 1.55 [0.68, 3.56]). NASH with any fibrosis stage was trending towards significance in PCE (OR [95% CI] for PCE: 1.66 [0.96, 2.86] and for FRS: 1.23 [0.58, 2.58]) (Table 2). Results were similar in sensitivity analyses for the PCE that restricted to NH Black and White participants aged ≥40 years: point estimates were comparable, but not all associations were statistically significant due to a decrease in sample size.

**Table 2:** Association of NASH and estimated fibrosis stages with elevated predicted CV risk, in adults aged ≥30 and matched to non-NASH controls.

	PCE		FRS	
	Weighted OR	P-value	Weighted OR	P-value
	(95% CI)		(95% CI)	
**Non-NASH**	1.0 (Ref)		1.0 (Ref)	
**(n = 2585)**				
**NASH**	1.66	0.07	1.23	0.57
**(n = 125)**	(0.96, 2.86)		(0.58, 2.58)	
**Non-NASH**	1.0 (Ref)		1.0 (Ref)	
**(n = 2585)**				
**NASH with no/minimal fibrosis**	0.31	0.02	0.41	0.09
**(n = 30)**	(0.12, 0.81)		(0.15, 1.14)	
**NASH with significant fibrosis**	2.34	0.01	1.55	0.29
**(n = 95)**	(1.25, 4.36)		(0.68, 3.56)	

NASH and non-NASH controls matched on age, sex, race/ethnicity, and diabetes status. Odds ratios and corresponding 95% confidence intervals and p-values were obtained using weighted univariate logistic regression. Participants with estimated fibrosis stage of F2 or higher (i.e., LSM ≥8.2) were considered to have significant fibrosis, while estimated stages F0-F1 (i.e., LSM <8.2) were considered no/minimal fibrosis.

Analyses restricted to participants under 65 years of age showed similar results, but associations were of greater magnitude and statistically significant with the PCE. Compared to participants with no NASH, those with NASH and significant fibrosis had 3.04 times the odds of having elevated predicted CV risk as calculated using the PCE (95% CI: 1.43, 6.44) and 2.02 times the odds as calculated using the FRS (95% CI: 0.61, 6.68) (Table 3).

**Table 3:** Association of NASH and estimated fibrosis stages with elevated predicted CV risk, in adults aged 30–64 and matched to non-NASH controls.

	PCE		FRS	
	Weighted OR (95% CI)	P-value	Weighted OR (95% CI)	P-value
**Non-NASH**	1.0 (Ref)		1.0 (Ref)	
**(n = 2260)**				
**NASH**	2.14	0.03	1.58	0.39
**(n = 100)**	(1.08, 4.21)		(0.54, 4.65)	
**Non-NASH**	1.0 (Ref)		1.0 (Ref)	
**(n = 2260)**				
**NASH with no/minimal fibrosis**	0.36	0.02	0.53	0.14
(n = 26*)	(0.16, 0.80)		(0.22, 1.24)	
**NASH with significant fibrosis**	3.04	<0.01	2.02	0.24
**(n = 74)**	(1.43, 6.44)		(0.61, 6.68)	

* NHANES analytic guidelines recommend unweighted sample size ≥30 for reporting proportions, means, and variances; reliability of these estimates should be interpreted with caution.

NASH and non-NASH controls matched on age, sex, race/ethnicity, and diabetes status. Odds ratios and corresponding 95% confidence intervals and p-values were obtained using weighted univariate logistic regression. Participants with estimated fibrosis stage of F2 or higher (i.e., LSM ≥8.2) were considered to have significant fibrosis, while estimated stages F0-F1 (i.e., LSM <8.2) were considered no/minimal fibrosis.

Additional sensitivity analyses that evaluated 10-year predicted CV risk as a continuous variable using linear regression resulted in null associations in the overall cohort (S1 Table in S1 File). However, when restricting to participants <65 years of age, NASH with significant fibrosis was associated with statistically significant higher incremental predicted CV risk in FRS as compared with no NASH (PCE: 1.4% [0.00%, 3.20%] and FRS: 2.9% [0.18%, 5.68%]) (S2 Table in S2 File).

To explore potential for bias from sample non-response, an exploratory analysis was conducted to assess the impact of reweighting the analytic sample for differences in age, sex, and race/ethnicity. This was found to have minimal impact, with limited change in effect magnitudes, and no change in statistical significance of results.

## Discussion

In this analysis, NASH was associated with higher 10-year predicted CV risk independent of diabetes. This association was more notable and of greater magnitude in persons with more severe liver fibrosis (i.e., estimated stages F2-F4) and persons under 65 years of age. These findings are important given patients with NASH have been shown to have more rapid liver fibrosis progression compared to those with NAFL. A systematic review and meta-analysis found that one in five patients who progress could be considered rapid progressors (transition from stage 0 to stage 3–4 fibrosis within an average of six years) [40].

According to the AHA, there is currently limited US population-based data on CV risk in NASH, and CV risk associated with NASH has not been well quantified. Several previous studies have matched individuals with NAFLD/NASH to those without NAFLD/NASH to evaluate the association with CVD [18, 20, 21, 41]. These studies have generally matched on age and sex, with subsequent adjustment for CVD risk factors. Most reported an increased risk of CVD in patients with NAFLD/NASH when compared with those without. However, these studies consisted of varying populations (adults vs children/young adults; a variety of geographic locations, different definitions of NAFLD/NASH, and different outcome definitions (MACE [20], CVD [defined as ischemic heart disease or stroke], [18] acute MI/stroke, [41] coronary artery disease, [14] and right ventricular dysfunction [21]).

One advantage of this study is its use of established CV risk equations to evaluate the potential for elevated CV risk in NASH. The PCE and FRS remain standard CV risk equations used in clinical practice; but when the PCE or FRS (and most other) CV risk scores are applied to contemporary cohorts, the PCE and FRS have been shown to overestimate CV risk [35, 42]. There is variability across risk scores with regard to outcome definitions (and correspondingly, predicted risks), which can impact which patients are identified as high-risk and are targeted for preventive therapy [43]. The fact that associations were more frequently found in PCE analyses may show a higher association with ASCVD than with outcomes included in FRS but not PCE such as atrial fibrillation or heart failure. Additionally, the performance of CV risk prediction equations that have been developed in a general population has not been well-studied in NAFLD/NASH populations. A recent study evaluated the performance of the PCE in persons with and without NAFLD and found that discrimination was worse in NAFLD, and calibration was poor overall. Observed CV risk was higher than predicted CV risk in persons with NAFLD (i.e., predicted CV risk was underestimated), and vice versa for those without NAFLD [44]. If predicted CV risk is overestimated in non-NAFLD/non-NASH and underestimated in NAFLD/NASH, then it is possible that the association between NASH and CV risk is of greater magnitude than suggested in this study, as our outcome was predicted CV risk. Further, as the risk equations used in this study do not account for potential mediators of the relationship between NASH and CV risk, such as liver fibrosis [22–24] and ectopic fat, [1] estimated CV risk may be further underestimated.

An important consideration in our study was the inclusion of diabetes in the matching algorithm. Components of metabolic syndrome including obesity, hypertension, dyslipidemia, and insulin resistance/T2D are recognized to be pre-disposing conditions for NASH, [29] and these components are associated with CV risk [15–17]. However, these conditions may also mediate the association of NASH and CV risk along the causal pathway, as recent evidence suggests a bi-directional relationship between NAFLD and metabolic syndrome [13]. For this reason, some previous studies have not matched on diabetes. By matching on diabetes, we have ensured that we have removed any potential confounding related to the condition. Consequently, it is possible that we may have removed some of the indirect effects of NASH on predicted CV risk that is mediated by diabetes. However, provided that we still found an association, this suggests that an effect of NASH on predicted CV risk still remains, above and beyond that either confounded or mediated by diabetes.

Our study had several limitations. First, because VCTE measurements are only available in recent continuous NHANES cycles, we could not leverage data from earlier cycles, which resulted in limited sample size for the analysis, which may impact the reliability of estimates. We have indicated estimates that may be unreliable and should be interpreted with caution. Nonetheless, these results provide insight into a topic that warrants further study with larger samples; incorporating VCTE measurements collected in future cycles of NHANES may help address uncertainty in results of this analysis. A second limitation is that the cross-sectional nature of the data prevented validation of predicted CV risk (i.e., based on longitudinal observations of CV events). Third, there were potential limitations in determining NASH and fibrosis status through non-invasive tests rather than liver biopsy, although this approach is consistent with other recent studies [45]. In addition, certain variables in the analysis relied on self-reported data, which could result in misclassification. If these variables were under-reported, this analysis may have incorrectly included some participants with other liver diseases in the NASH group. Among participants identified as having presumed NAFLD, use of FAST score ≥0.48 to predict NASH should be interpreted in relation to the predictive accuracy (i.e., sensitivity, specificity, PPV, and NPV) described earlier. Fourth, currently, there are no consensus cutoffs for fibrosis stages as measured by VCTE in NAFLD/NASH [46, 47]. Fifth, given the small number of participants with NASH with no/minimal fibrosis (n = 30 in the main analysis and n = 26 in the analysis that restricts to adults aged 30–64 years), we refrain from interpreting this category as per NHANES analytic guidelines. In addition, limited sample size of NASH patients aged 65 and older (n = 25) prevents us from examining that subgroup in further detail. Although this research was conducted to align with the AHA statement, future studies could seek to confirm these findings in MASLD and MASH populations. Finally, although we concluded that NASH was associated with higher predicted CV risk independent of diabetes, this study cannot rule out potential residual confounding by other conditions commonly comorbid with NASH (e.g., obesity).

## Conclusion

In this study, a significant association between NASH and primary CV event risk was observed, independent of diabetes, particularly in adults between 30 and 64 years of age. The incremental risk was greater for estimated fibrosis stages F2-F4, suggesting the importance of accounting for fibrosis stage in predicting CV risk. This analysis suggests that NASH with significant fibrosis confers increased CV risk above that associated with diabetes.

## Supporting information

S1 File Results of continuous predicted CV risk in matched adults aged ≥30. (DOCX)

S2 File Results of continuous predicted CV risk in matched adults aged 30–64. (DOCX)

## References

[1] Duell PB, Welty FK, Miller M, Chait A, Hammond G, Ahmad Z, et al. Nonalcoholic Fatty Liver Disease and Cardiovascular Risk: A Scientific Statement From the American Heart Association. Arteriosclerosis, thrombosis, and vascular biology. 2022;42(6):e168–e85. doi: 10.1161/ATV.0000000000000153 35418240

[2] Sheth SG, Chopra S. Epidemiology, clinical features, and diagnosis of nonalcoholic fatty liver disease in adults. UpToDate.

[3] American Liver Foundation. Nonalcoholic Fatty Liver Disease (NAFLD). URL: https://liverfoundation.org/for-patients/about-the-liver/diseases-of-the-liver/non-alcoholic-fatty-liver-disease/.

[4] Armandi A, Bugianesi E. Natural history of NASH. Liver Int. 2021;41 Suppl 1(Suppl 1):78–82. doi: 10.1111/liv.14910 34155792 PMC8361694

[5] Kanwal F, Shubrook JH, Younossi Z, Natarajan Y, Bugianesi E, Rinella ME, et al. Preparing for the NASH epidemic: A call to action. Metabolism: clinical and experimental. 2021;122:154822. doi: 10.1016/j.metabol.2021.154822 34289945

[6] Diehl AM, Day C. Cause, Pathogenesis, and Treatment of Nonalcoholic Steatohepatitis. N Engl J Med. 2017;377(21):2063–72. doi: 10.1056/NEJMra1503519 29166236

[7] Rich NE, Oji S, Mufti AR, Browning JD, Parikh ND, Odewole M, et al. Racial and Ethnic Disparities in Nonalcoholic Fatty Liver Disease Prevalence, Severity, and Outcomes in the United States: A Systematic Review and Meta-analysis. Clin Gastroenterol Hepatol. 2018;16(2):198–210.e2. doi: 10.1016/j.cgh.2017.09.041 28970148 PMC5794571

[8] Younossi ZM, Blissett D, Blissett R, Henry L, Stepanova M, Younossi Y, et al. The economic and clinical burden of nonalcoholic fatty liver disease in the United States and Europe. Hepatology. 2016;64(5):1577–86. doi: 10.1002/hep.28785 27543837

[9] Hamid O, Eltelbany A, Mohammed A, Alsabbagh Alchirazi K, Trakroo S, Asaad I. The epidemiology of non-alcoholic steatohepatitis (NASH) in the United States between 2010–2020: a population-based study. Annals of hepatology. 2022;27(5):100727. doi: 10.1016/j.aohep.2022.100727 35700934

[10] Williams CD, Stengel J, Asike MI, Torres DM, Shaw J, Contreras M, et al. Prevalence of nonalcoholic fatty liver disease and nonalcoholic steatohepatitis among a largely middle-aged population utilizing ultrasound and liver biopsy: a prospective study. Gastroenterology. 2011;140(1):124–31. doi: 10.1053/j.gastro.2010.09.038 20858492

[11] Estes C, Razavi H, Loomba R, Younossi Z, Sanyal AJ. Modeling the epidemic of nonalcoholic fatty liver disease demonstrates an exponential increase in burden of disease. Hepatology. 2018;67(1):123–33. doi: 10.1002/hep.29466 28802062 PMC5767767

[12] American Liver Foundation. Nash Complications. URL: https://liverfoundation.org/for-patients/about-the-liver/diseases-of-the-liver/nonalcoholic-steatohepatitis-information-center/nash-complications/.

[13] Targher G, Byrne CD, Lonardo A, Zoppini G, Barbui C. Non-alcoholic fatty liver disease and risk of incident cardiovascular disease: A meta-analysis. Journal of hepatology. 2016;65(3):589–600. doi: 10.1016/j.jhep.2016.05.013 27212244

[14] Beer S, Babel J, Martin N, Blank V, Wiegand J, Karlas T. Non-invasive assessment of steatohepatitis indicates increased risk of coronary artery disease. Plos one. 2023;18(9):e0286882. doi: 10.1371/journal.pone.0286882 37768969 PMC10538770

[15] Targher G, Byrne CD. Clinical Review: Nonalcoholic fatty liver disease: a novel cardiometabolic risk factor for type 2 diabetes and its complications. J Clin Endocrinol Metab. 2013;98(2):483–95. doi: 10.1210/jc.2012-3093 23293330

[16] Goff DC, Jr., Lloyd-Jones DM, Bennett G, Coady S, D’Agostino RB, Gibbons R, et al. 2013 ACC/AHA guideline on the assessment of cardiovascular risk: a report of the American College of Cardiology/American Heart Association Task Force on Practice Guidelines. Circulation. 2014;129(25 Suppl 2):S49–73. doi: 10.1161/01.cir.0000437741.48606.98 24222018

[17] D’Agostino RB, Sr., Vasan RS, Pencina MJ, Wolf PA, Cobain M, Massaro JM, et al. General cardiovascular risk profile for use in primary care: the Framingham Heart Study. Circulation. 2008;117(6):743–53. doi: 10.1161/CIRCULATIONAHA.107.699579 18212285

[18] Hagstrom H, Nasr P, Ekstedt M, Hammar U, Stal P, Askling J, et al. Cardiovascular risk factors in non-alcoholic fatty liver disease. Liver Int. 2019;39(1):197–204. doi: 10.1111/liv.13973 30253056

[19] Hussain A, Nambi V, Selvin E, Sun W, Matsushita K, Yu B, et al. Abstract 14043: Association of Non-Alcoholic Steatohepatitis Assessed by Fib-4 Index and Risk of Cardiovascular Disease: The Atherosclerosis Risk in Communities (ARIC) Study. Circulation. 2020;142(Suppl_3):A14043-A. doi: 10.1161/circ.142.suppl_3.14043

[20] Simon TG, Roelstraete B, Alkhouri N, Hagstrom H, Sundstrom J, Ludvigsson JF. Cardiovascular disease risk in paediatric and young adult non-alcoholic fatty liver disease. Gut. 2023;72(3):573–80. doi: 10.1136/gutjnl-2022-328105 36522149

[21] Sunbul M, Kivrak T, Durmus E, Akin H, Aydin Y, Ergelen R, et al. Nonalcoholic Steatohepatitis Score is an Independent Predictor of Right Ventricular Dysfunction in Patients with Nonalcoholic Fatty Liver Disease. Cardiovasc Ther. 2015;33(5):294–9. doi: 10.1111/1755-5922.12145 26202098

[22] Athyros VG, Tziomalos K, Katsiki N, Doumas M, Karagiannis A, Mikhailidis DP. Cardiovascular risk across the histological spectrum and the clinical manifestations of non-alcoholic fatty liver disease: An update. World J Gastroenterol. 2015;21(22):6820–34. doi: 10.3748/wjg.v21.i22.6820 26078558 PMC4462722

[23] Kim D, Kim WR, Kim HJ, Therneau TM. Association between noninvasive fibrosis markers and mortality among adults with nonalcoholic fatty liver disease in the United States. Hepatology. 2013;57(4):1357–65. doi: 10.1002/hep.26156 23175136 PMC3622816

[24] Ekstedt M, Hagstrom H, Nasr P, Fredrikson M, Stal P, Kechagias S, et al. Fibrosis stage is the strongest predictor for disease-specific mortality in NAFLD after up to 33 years of follow-up. Hepatology. 2015;61(5):1547–54. doi: 10.1002/hep.27368 25125077

[25] National Center for Health Statistics. NHANES Analytic Guidance and Brief Overview for the 2017-March 2020 Pre-pandemic Data Files. Last updated: June 22, 2021. URL: https://wwwn.cdc.gov/nchs/nhanes/continuousnhanes/OverviewBrief.aspx?Cycle=2017-2020.

[26] National Center for Health Statistics. NCHS Research Ethics Review Board (ERB) Approval [Internet]. Available at: https://www.cdc.gov/nchs/nhanes/irba98.htm.

[27] Kabbany MN, Conjeevaram Selvakumar PK, Watt K, Lopez R, Akras Z, Zein N, et al. Prevalence of Nonalcoholic Steatohepatitis-Associated Cirrhosis in the United States: An Analysis of National Health and Nutrition Examination Survey Data. Am J Gastroenterol. 2017;112(4):581–7. doi: 10.1038/ajg.2017.5 28195177

[28] Younossi ZM, Stepanova M, Afendy M, Fang Y, Younossi Y, Mir H, et al. Changes in the prevalence of the most common causes of chronic liver diseases in the United States from 1988 to 2008. Clin Gastroenterol Hepatol. 2011;9(6):524–30.e1; quiz e60. doi: 10.1016/j.cgh.2011.03.020 21440669

[29] Rinella ME, Lazarus JV, Ratziu V, Francque SM, Sanyal AJ, Kanwal F, et al. A multisociety Delphi consensus statement on new fatty liver disease nomenclature. Journal of Hepatology. 2023;79(6):1542–56. doi: 10.1016/j.jhep.2023.06.003 37364790

[30] Lee JS, Lee HW, Kim BK, Park JY, Kim DY, Ahn SH, et al. Comparison of FibroScan-Aspartate Aminotransferase (FAST) Score and Other Non-invasive Surrogates in Predicting High-Risk Non-alcoholic Steatohepatitis Criteria. Front Med (Lausanne). 2022;9:869190. doi: 10.3389/fmed.2022.869190 35492369 PMC9048204

[31] Newsome PN, Sasso M, Deeks JJ, Paredes A, Boursier J, Chan WK, et al. FibroScan-AST (FAST) score for the non-invasive identification of patients with non-alcoholic steatohepatitis with significant activity and fibrosis: a prospective derivation and global validation study. Lancet Gastroenterol Hepatol. 2020;5(4):362–73. doi: 10.1016/S2468-1253(19)30383-8 32027858 PMC7066580

[32] Eddowes PJ, Sasso M, Allison M, Tsochatzis E, Anstee QM, Sheridan D, et al. Accuracy of FibroScan Controlled Attenuation Parameter and Liver Stiffness Measurement in Assessing Steatosis and Fibrosis in Patients With Nonalcoholic Fatty Liver Disease. Gastroenterology. 2019;156(6):1717–30. doi: 10.1053/j.gastro.2019.01.042 30689971

[33] Flores Rosario K, Mehta A, Ayers C, Engel Gonzalez P, Pandey A, Khera R, et al. Performance of the Pooled Cohort Equations in Hispanic Individuals Across the United States: Insights From the Multi-Ethnic Study of Atherosclerosis and the Dallas Heart Study. J Am Heart Assoc. 2021;10(9):e018410. doi: 10.1161/JAHA.120.018410 33870702 PMC8200750

[34] Rodriguez F, Chung S, Blum MR, Coulet A, Basu S, Palaniappan LP. Atherosclerotic Cardiovascular Disease Risk Prediction in Disaggregated Asian and Hispanic Subgroups Using Electronic Health Records. J Am Heart Assoc. 2019;8(14):e011874. doi: 10.1161/JAHA.118.011874 31291803 PMC6662141

[35] Damen JA, Pajouheshnia R, Heus P, Moons KGM, Reitsma JB, Scholten R, et al. Performance of the Framingham risk models and pooled cohort equations for predicting 10-year risk of cardiovascular disease: a systematic review and meta-analysis. BMC Med. 2019;17(1):109. doi: 10.1186/s12916-019-1340-7 31189462 PMC6563379

[36] Friedewald WT, Levy RI, Fredrickson DS. Estimation of the concentration of low-density lipoprotein cholesterol in plasma, without use of the preparative ultracentrifuge. Clin Chem. 1972;18(6):499–502. 4337382

[37] Sterling RK, Lissen E, Clumeck N, Sola R, Correa MC, Montaner J, et al. Development of a simple noninvasive index to predict significant fibrosis in patients with HIV/HCV coinfection. Hepatology. 2006;43(6):1317–25. doi: 10.1002/hep.21178 16729309

[38] Greifer N. MatchIt (R package)—Assessing Balance [Internet]. June 13, 2023. URL: https://cran.r-project.org/web/packages/MatchIt/vignettes/assessing-balance.html.

[39] Stone NJ, Robinson JG, Lichtenstein AH, Bairey Merz CN, Blum CB, Eckel RH, et al. 2013 ACC/AHA guideline on the treatment of blood cholesterol to reduce atherosclerotic cardiovascular risk in adults: a report of the American College of Cardiology/American Heart Association Task Force on Practice Guidelines. Circulation. 2014;129(25 Suppl 2):S1–45. doi: 10.1161/01.cir.0000437738.63853.7a 24222016

[40] Singh S, Allen AM, Wang Z, Prokop LJ, Murad MH, Loomba R. Fibrosis progression in nonalcoholic fatty liver vs nonalcoholic steatohepatitis: a systematic review and meta-analysis of paired-biopsy studies. Clin Gastroenterol Hepatol. 2015;13(4):643–54 e1-9; quiz e39-40. doi: 10.1016/j.cgh.2014.04.014 24768810 PMC4208976

[41] Alexander M, Loomis AK, van der Lei J, Duarte-Salles T, Prieto-Alhambra D, Ansell D, et al. Non-alcoholic fatty liver disease and risk of incident acute myocardial infarction and stroke: findings from matched cohort study of 18 million European adults. BMJ. 2019;367:l5367. doi: 10.1136/bmj.l5367 31594780 PMC6780322

[42] DeFilippis AP, Young R, Carrubba CJ, McEvoy JW, Budoff MJ, Blumenthal RS, et al. An analysis of calibration and discrimination among multiple cardiovascular risk scores in a modern multiethnic cohort. Ann Intern Med. 2015;162(4):266–75. doi: 10.7326/M14-1281 25686167 PMC4414494

[43] Lagerweij GR, Moons KGM, de Wit GA, Koffijberg H. Interpretation of CVD risk predictions in clinical practice: Mission impossible? PLoS One. 2019;14(1):e0209314. doi: 10.1371/journal.pone.0209314 30625177 PMC6326414

[44] Henson JB, Budoff MJ, Muir AJ. Performance of the Pooled Cohort Equations in non-alcoholic fatty liver disease: The Multi-Ethnic Study of Atherosclerosis. Liver Int. 2023;43(3):599–607. doi: 10.1111/liv.15480 36401810 PMC9974541

[45] Thévenot T, Vendeville S, Weil D, Akkouche L, Calame P, Canivet CM, et al. Systematic screening for advanced liver fibrosis in patients with coronary artery disease: The CORONASH study. Plos one. 2022;17(5):e0266965. doi: 10.1371/journal.pone.0266965 35617294 PMC9135299

[46] Boursier J, Vergniol J, Guillet A, Hiriart JB, Lannes A, Le Bail B, et al. Diagnostic accuracy and prognostic significance of blood fibrosis tests and liver stiffness measurement by FibroScan in non-alcoholic fatty liver disease. J Hepatol. 2016;65(3):570–8. doi: 10.1016/j.jhep.2016.04.023 27151181

[47] Patel K, Sebastiani G. Limitations of non-invasive tests for assessment of liver fibrosis. JHEP Rep. 2020;2(2):100067. doi: 10.1016/j.jhepr.2020.100067 32118201 PMC7047178

