# Supplementary material for: Cardiovascular risk in US adults with nonalcoholic steatohepatitis (NASH) vs. matched non-NASH controls, National Health and Nutrition Examination Survey, 2017–2020
Source: PLoS One. 2024 Aug 27;19(8):e0309617. doi: 10.1371/journal.pone.0309617 (PMC11349199; doi:10.1371/journal.pone.0309617)
Supplement: S1 File — (DOCX) [file pone.0309617.s001.docx]

**Supporting Information**

**S1 File –** **Results of continuous predicted CV risk in matched adults aged ≥30**

**Table S1.** Association between NASH and estimated fibrosis stages with continuous predicted CV risk, in adults aged ≥30 and matched to non-NASH controls

|  | **PCE** | | **FRS** | |
| --- | --- | --- | --- | --- |
|  | **Weighted coef.**  **(SE)** | **P-value** | **Weighted coef.**  **(SE)** | **P-value** |
| **Non-NASH**  **(n=2585)** | 6.24 (Ref) |  | 10.98 (Ref) |  |
| **NASH**  **(n = 125)** | 0.30  (1.06) | 0.78 | 1.40  (1.39) | 0.32 |
| **Non-NASH**  **(n=2585)** | 6.24 (Ref) |  | 10.98 (Ref) |  |
| **NASH with no/minimal fibrosis**  **(n=30)** | -2.22  (0.97) | 0.03 | -2.09  (1.52) | 0.18 |
| **NASH with significant fibrosis**  **(n=95)** | 1.14  (1.29) | 0.39 | 2.56  (1.68) | 0.14 |

NASH and non-NASH controls matched on age, sex, race/ethnicity, and diabetes status. Weighted univariate linear regression was used to obtain beta coefficients and corresponding standard errors (SEs) and p-values. The intercept value reflects the mean predicted CV risk for non-NASH; NASH coefficients are additive to the intercept to calculate absolute mean probability.
