# Supplementary material for: Cardiovascular risk in US adults with nonalcoholic steatohepatitis (NASH) vs. matched non-NASH controls, National Health and Nutrition Examination Survey, 2017–2020
Source: PLoS One. 2024 Aug 27;19(8):e0309617. doi: 10.1371/journal.pone.0309617 (PMC11349199; doi:10.1371/journal.pone.0309617)
Supplement: S2 File — (DOCX) [file pone.0309617.s002.docx]

**Supporting Information**

**S2 File – Results of continuous predicted CV risk in matched adults aged 30-64**

**Table S2. Association between NASH and estimated fibrosis stages with continuous predicted CV risk, in adults aged 30-64 and matched to non-NASH controls**

|  | **PCE** | | **FRS** | |
| --- | --- | --- | --- | --- |
|  | **Weighted coef.**  **(SE)** | **P-value** | **Weighted coef.**  **(SE)** | **P-value** |
| **Non-NASH**  **(n=2260)** | 3.79 (Ref) |  | 8.14 (Ref) |  |
| **NASH**  **(n=100)** | 0.82  (0.69) | 0.24 | 2.01  (1.09) | 0.08 |
| **Non-NASH**  **(n=2260)** | 3.79 (Ref) |  | 8.14 (Ref) |  |
| **NASH with no/minimal fibrosis**  **(n=26*)** | -0.82  (0.60) | 0.19 | -0.48  (1.30) | 0.71 |
| **NASH with significant fibrosis**  **(n=74)** | 1.43  (0.86) | 0.11 | 2.93  (1.33) | 0.04 |

* NHANES analytic guidelines recommend unweighted sample size ≥30 for reporting proportions, means, and variances; reliability of these estimates should be interpreted with caution.

NASH and non-NASH controls matched on age, sex, race/ethnicity, and diabetes status. Weighted univariate linear regression was used to obtain beta coefficients and corresponding standard errors (SEs) and p-values. The intercept value reflects the mean predicted CV risk for non-NASH; NASH coefficients are additive to the intercept to calculate absolute mean probability.
